# Therapeutic mechanism of *Pithecellobium clypearia* Benth. on imiquimod-induced psoriasis revealed by tissue transcriptomics in mice

**DOI:** 10.1371/journal.pone.0333197

**Published:** 2025-12-12

**Authors:** Xiyuan He, Yueting Mo, Peixin Shi, Yini Xu, Mingmei Zhou, Ting Zhang

**Affiliations:** 1 Institute of Interdisciplinary Integrative Medicine Research, Shanghai University of Traditional Chinese Medicine, Shanghai, China; 2 State Key Laboratory of Functions and Applications of Medicinal Plants, Guizhou Medical University, Guiyang, China; Kansai Medical University: Kansai Ika Daigaku, Institute of Biomedical Science, JAPAN

## Abstract

**Background:**

Psoriasis is an erythema papulosquamous dermatosis that cannot be cured at present. *Pithecellobium clypearia* Benth. belonging to the Leguminosae family and is clinically used as a treatment for gastroenteritis, acute tonsillitis, acute pharyngitis, and upper respiratory tract infections. Our previous studies have found that *P. clypearia* can improve imiquimod (IMQ)-induced psoriasis in mice and have revealed some differential metabolites and pathways using metabolomics methods. However, the underlying molecular mechanisms remain obscure. The purpose of this study is to investigate the therapeutic mechanism of the anti-psoriatic effects of *P. clypearia* using transcriptomics technology.

**Methods:**

The psoriasis model was induced in male Balb/c mice by applying IMQ on their backs. To identify the differentially expressed genes (DEGs) among groups, RNA sequencing was employed. DEGs were analyzed using Gene Ontology (GO), Kyoto Encyclopedia of Genes and Genomes (KEGG) pathway analysis, and protein-protein interaction (PPI) network analysis. Furthermore, quantitative real-time PCR (qPCR) was employed for validation of these results.

**Results:**

A total of 26 DEGs were identified, with several enriched pathways, including the MAPK signaling pathway, unfolded proteins response, hedgehog signaling pathways, NADH dehydrogenase activity, oxidative phosphorylation. Additionally, PPI network analysis revealed that gene *Hspa1a* was connected with *Hspa1b*, *Bcl2* and *GzmA*, and *Asns* was related to *Trib3*, *Slc7a5* and *Chac1*, and *mt-Nd4l* was correlated with *mt-Nd5* and *mt-Nd6*. The RNA-seq results were concordant with the qPCR results.

**Conclusions:**

*P. clypearia* may ameliorate inflammation in psoriasis mice by modulating genes such as *Hspa1a*, *Hspa1b*, *mt-Nd4l*, *mt-Nd5*, *mt-Nd6*, *Bcl2*, *Asns*, *Trib3*, and associated pathways related to energy metabolism, cell growth, and apoptosis. Our study explored the underlying molecular mechanisms at the transcriptome level and provided a theoretical basis for further investigation into the efficacy of *P. clypearia*.

## Introduction

Psoriasis is a chronic inflammatory skin condition characterized by a strong genetic predisposition and autoimmune nature. Its etiology is multifactorial, with hallmark clinical manifestations including sharply defined, erythematous plaques that are both itchy and covered with silvery scales. Current understanding of the psoriasis pathogenesis involves genetic susceptibility, environmental triggers, and IL-23/IL-17 axis. Various treatments are available for different disease severities, including glucocorticoids, vitamin D analogs, phototherapy, and targeted biological drugs [1]. Despite these advancements, psoriasis remains a treatable but incurable condition. The long-term treatment process not only imposes significant physical and financial burdens on patients but also profoundly impacts their quality of life. Individuals with psoriasis are more likely to suffer from depression (up to 20%) and even suicidal compared to the general population. There is an urgent need for anti-psoriasis drugs that are both highly effective and safe.

Traditional Chinese Medicine (TCM) shows potential on treating psoriasis due to low toxicity, high efficiency, prevention of recurrence and low cost, with the treatment principles of clearing heat and detoxifying, promoting blood circulation and removing blood stasis, and nourishing blood. Furthermore, according to the theory of TCM that the lung governs the skin and fur, the lung can promote the distribution of vital energy and body fluids to maintain the skin barrier, warming and nourishing the skin and fur, while the fur can promote the dispersion of lung qi and help the lung breathe. Previous studies have demonstrated that adolescents often experience upper respiratory tract infections or acute tonsillitis prior to the onset of psoriasis [2,3]. Psoriasis patients often have a history of upper respiratory tract infection during cooler weather or dry autumn and winter. In some cases, tonsillectomy is performed to alleviate refractory psoriatic symptoms when conventional treatments fail, highlighting the need for targeted pharmacological interventions. Thus, some herbal medicines with treating respiratory tract infections, clearing lung heat and nourishing the lung are often used in the treatment of psoriasis [4]. Additionally, the structural diversity and multi-target mechanisms of TCM’s chemical constituents enable synergistic activity in alleviating psoriasis [5]. Several herbs and their components have demonstrated efficacy in treating psoriasis, such as *Cimicifuga foetida L.*, *Rheum palmatum* L. [6,7].

*Pithecellobium clypearia* Benth, a traditional folk medicine used in TCM, has been documented for its anti-inflammatory and immune regulation effects since Li Shizhen’s Compendium of Materia Medica during the Ming Dynasty (1390 A.D.). It is commonly used in clinical practice to treat bacterial dysentery, acute tonsillitis, acute gastroenteritis, pharyngitis, laryngitis, and upper respiratory tract infections [8]. *P. clypearia* is known for its ability to detoxify and clear heat, cool blood, reduce edema, and stop diarrhea. Phytochemical and pharmacological studies have identified various components in *P. clypearia*, including flavonoids, phenylpropanoids, organic phenolic acids, triterpenoids, and steroids [8], which exhibit anti-bacterial, anti-cancer, anti-oxidative, anti-inflammation activities, as well as immunological function [9–11]. Moreover, the traditional efficacy of *P. clypearia* aligns with the TCM principles for psoriasis and the TCM theory that the lungs govern the skin and fur, suggesting its potential as a therapeutic agent for this condition.

In our previous study, the toxicity and dosing regimen of the ethanol supernatant of water extract (ESW) from *P. clypearia* were preliminarily evaluated. The findings revealed no toxic reactions at a dose of 27 g/kg, which exceeded the clinically relevant human dose. Over a 14-day observation period, no mortality was observed among the mice, and no abnormalities were detected in anatomical organs upon dissection. These results collectively suggested that ESW was safe for use under the tested conditions. Moreover, after ESW administration in IMQ-induced psoriasis mice, significant reductions were observed in PASI score, epidermal thickness, parakeratosis, epidermal hyperplasia, lymphocyte infiltration, and spleen index compared to untreated controls. Metabolomics studies showed that ESW modulated 17 biomarkers associated with 21 metabolic pathways in spleen samples and 6 biomarkers associated with 11 pathways in serum samples, thereby ameliorating psoriatic lesions. The most affected spleen functions included phenylalanine, tyrosine, and tryptophan biosynthesis, and serine, threonine, starch, and sucrose metabolism. Serum samples exhibited significant enrichment in galactose, phosphatidylinositol, and starch/sucrose metabolism pathways [12]. These results further confirmed that *P. clypearia* could effectively relieve psoriasis skin inflammation.

Transcriptomics has emerged as a powerful tool for analyzing global transcriptional activity, with applications extending to the identification of novel therapeutic targets in complex diseases such as psoriasis. RNA-seq, the most common transcriptomics technique, provides advantages such as a wide detection range, high throughput, and good reproducibility. Due to the multi-component and multi-target characteristics of TCM, omics research provides strong support for its general principles and dialectical approaches. In this study, we conducted transcriptomic analysis on mice spleen tissues to elucidate the mechanism by which *P. clypearia* alleviates IMQ-induced psoriasis-like lesions at the genetic level.

## Materials and methods

### Materials and reagents

TRIzol^®^ Reagent was obtained from Invitrogen (USA). Truseq^TM^ RNA Sample Preparation Kit, HiSeq 4000 SBS Kit (300 cycles) and transcriptome sequencing primers were purchased from Illumina (USA). DNase I endonuclease and agarose gel were supplied by TaKaRa (Japan). PCR primers were obtained from Hongxun Technology Biotechnology Co., Ltd. (Suzhou). The 2X Taq Plus Master was provided by Novozan Biotechnology Co., Ltd. (Nanjing), and the DL2000 was sourced from Shanghai Jierui Biological Engineering Co., Ltd. (Shanghai).

### Preparation of extracts

The *P. clypearia* twigs and leaves were supplied by Hutchison Whampoa Guangzhou Baiyunshan Chinese Medicine Co., Ltd. t and authenticated by Professor Ting Zhang. The voucher specimen (No. 170719) was deposited at the Institute of Interdisciplinary Integrative Medicine Research Laboratory at Shanghai University of Traditional Chinese Medicine, Shanghai, China. The ESW was prepared in our laboratory [12].

### Animals

Male Balb/c mice, weighing between 18 and 22 grams, were provided by Shanghai SLAC Laboratory Animal Co. Ltd. All animal experiments had obtained written approval from the Ethics Committee for Animal Experiments of Shanghai University of Traditional Chinese Medicine. The mice were housed under a 12-hour light/12-hour dark cycle, with a temperature of 23 ± 3 °C and a relative humidity of 40–70%. They were allowed free access to standard laboratory chow and water for one week.

### Model preparation and drug application

The model group (IMQ group) and the ESW treatment group (ESW group) received 42 mg of commercially available 5% w/w IMQ cream (Mingxin Pharmaceuticals, Sichuan, China) daily after the backs were shaved (2 cm × 3 cm). The control group (Con group) received Vaseline on their backs. IMQ group and Con group were administered the vehicle (ddH2O, 400 μL). ESW group was treated with ESW (2.4 g/kg). The experiment lasted for 7 days.

### Experimental sample collection

On the seventh day of the experiment, all animals were euthanized via intraperitoneal injection of 1% pentobarbital sodium at a dose of 50 mg/kg body weight. Deep anesthesia and absence of pedal reflex were confirmed prior to tissue collection. Spleen tissues were immediately excised, snap-frozen in liquid nitrogen, and stored at −80 °C until further analysis. Every effort was made to minimize animal suffering, including continuous monitoring for signs of distress, confirmation of complete anesthesia prior to tissue collection, and rapid tissue harvesting to reduce procedure time.

### Total RNA isolation, library building and high-throughput sequencing

Total RNA was extracted from the spleen tissues of the Con, IMQ and ESW group using Trizol Reagent. The quality of RNA was assessed using the Agilent Bioanalyzer (Agilent Technologies, Inc.), which provided RNA integrity numbers ranging from 1 and 10. Then, poly-A in eukaryotic mRNA was selectively captured and enriched using Oligo (dT) beads. The enriched mRNA was split by fragmentation buffer into short pieces, and transcribed into cDNA by random hexamers. Finally, the fragmented cDNA was connected with sequence adapters by adding End Repair Mix for further sequencing analysis. For every splenic tissue, RNA libraries were created. The ligation products were selected by using agarose gel electrophoresis, PCR amplification, and Illumina Hi-Seq 4000 sequencing (Illumina, San Diego, California, USA).

### Raw data processing and identification of DEGs

The raw reads were pre-processed to ensure data availability by removing low-quality reads from the 3′ end, discarding low-quality reads with less than 50 of the quality value, sequences shorter than 20 bp, and raw reads with adapters or more than 10% of the N ratio. Quality metrics, including clean bases, GC content (%), Q20 and Q30 values (%), clean readings, and the error rate (%) were assessed. High-quality reads were mapped to the reference Mus-musculus genome (GRCm38) (http://asia.ensembl.org/Mus_musculus/Info/Index) using Hisat2 (HISAT2), and unique mapped reads were retrieved. The unique mapped reads were quantified and normalized using the FPKM (Fragments Per Kilobase of transcript per Million mapped reads) method and the program RSEM in order to evaluate the gene expression levels. The DEGs were then identified using the DESeq2 1.10.1 program, and the comparison’s settings were adjusted to *P* < 0.05 and |log2FC| > 1 for significant DEGs.

### GO functions and KEGG pathways analysis of DEGs

Every DEG identified in splenic tissue was annotated using KEGG and GO analysis for functional annotation and classification. Specifically, GO analysis was employed to categorize DEGs into hierarchical classifications for elucidating the genetic regulatory networks. These classifications encompassed three main domains: biological process (BP), cellular component (CC), and molecular function (MF). Additionally, KEGG pathway analysis was performed to identify significant pathways associated with DEGs. With default settings, the software tools BLAST2GO 2.5.0 and KOBAS 2.1.2 were able to obtain GO annotations and the KEGG pathway.

### Quantitative real-time PCR validation

To further validate the findings of RNA-seq, qPCR was employed to verify the expression of genes: *mt-Nd5*, *mt-Nd6*, *GzmA, CTSG*. Reverse transcription was performed using 1 μg of total RNA, with 18S rRNA serving as a reference gene. qPCR was performed with the MG96 + PCR System (Hangzhou Langji Scientific Instrument Co., Ltd., China). The reaction mixture consisted of 16.5 µL of ChamQ SYBR Color qPCR Master Mix (2×), 0.8 μL of forward and reverse primers (5 μmol/L), and 2 μL of template cDNA. The qPCR conditions included an initial denaturation for 5 seconds at 95°C, annealing for 30 seconds at 55°C, and extension for 40 seconds at 72°C, followed by a 40-cycle reaction. Primers used for all tested genes were listed in Table 1.

**Table 1:** Sequences of primers used for qPCR analysis.

Gene name	Primer Type	5′- 3′
*mt-Nd5*	F	TATAAMFGCATCGGAGAC
*mt-Nd5*	R	TGGTAGTCATGGGTGGAG
*mt-Nd6*	F	GAGGTTGATGATGTTGGAGT
*mt-Nd6*	R	AAATACCCGCAAACAAAG
*GzmA*	F	TGAAAGAATCATTGGAGGAG
*GzmA*	R	GTTACAGTGGGCAGCAGT
*CTSG*	F	GGGCTGAGTGCTTGTGGA
*CTSG*	R	CGGATGTTCTGCGGATTG

### PPI network construction

To investigate gene interactions, the PPI network of DEGs was constructed using the STRING database(http://string-db.org). Interactions with a combined score greater than 0.4 were taken into consideration.

### Statistical analysis

All experiments were duplicated at least thrice, and the corresponding results were showed as means ± SD. Data analysis was done using SPSS software. A difference was deemed statistically significant if the *P* value was less than 0.05, and highly significant if it was less than 0.01.

## Results

### Ameliorative Effects of ESW on IMQ-Induced Psoriasis-like Skin Lesions in Animal Models

**Fig 1:**
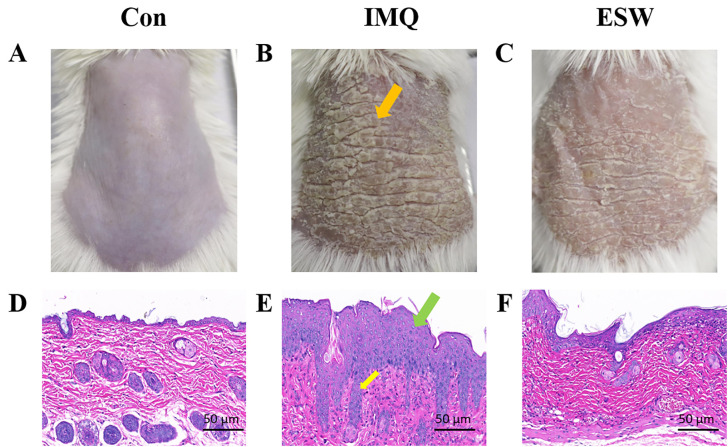
Representative images and pathological sections (Hematoxylin and Eosin Staining, 40×) of the back skins of Con, IMQ, ESW groups. In the figure, A, B and C were the back skin images of Con, IMQ and ESW groups, while D, E and F were the representative HE images of Con, IMQ and ESW groups.

As shown in Fig 1B and 1E, IMQ-induced psoriasis presents with pronounced scaling and hyperkeratosis (indicated by orange arrows) compared to Con group depicted in Fig 1A and 1D. Histological examination revealed the thickened epidermis characterized by elongation of the rete ridges and significant infiltration of inflammatory cells in the dermis (indicated by green and yellow arrows). Administration of ESW significantly reversed these conditions, as demonstrated in Fig 1C and 1F.

### RNA sequencing results and global gene expression profiles

RNA high-throughput sequencing was conducted by isolating RNA extracts from the spleen in Con, IMQ and ESW groups to identify the genes and pathways associated with the beneficial effects of ESW in psoriasis mice. Six RNA libraries in all were created during the sequencing process.

Sequencing data presented in Table 2 indicated that every library had acquired more than 43.5 million raw reads. Beyond 43.0 million clean reads were generated by removing the reads which included adapter and low quality combined with poly A during stringent filtering. Moreover, Q20 ratio for each library’s clean data exceeded greater than 97.27%, and the GC contents of each sample’s clean data ranged between 50.5% and 51.74%. Approximately 94.05 to 95.39% of the clean, high-quality reads were effectively mapped to the reference GRCm38. Furthermore, the unique mapping ratios for the splenic tissues from the Con, IMQ, and ESW groups ranged from 83.85 to 87.68%.

**Table 2:** Summary of sequencing reads and mapping results generated from Con, IMQ, and ESW groups.

Samples	Con-1	Con-2	IMQ-1	IMQ-2	ESW-1	ESW-2
Raw reads	43576952	52256282	57369162	43869114	46503422	49917606
Raw bases	6580119752	7890698582	8662743462	6624236214	7022016722	7537558506
Error rate of raw data (%)	0.0283	0.0276	0.0273	0.0274	0.028	0.0277
Q20 of raw data (%)	96.53	96.83	96.97	96.9	96.68	96.82
GC of raw data (%)	51.34	50.79	51.75	51.73	50.53	51.23
Clean reads	43001618	51683604	56884578	43412048	46062734	49493736
Clean bases	6394693720	7699426555	8471744527	6461190466	6859893614	7387385939
Error rate ofclean data (%)	0.0269	0.0265	0.0264	0.0263	0.027	0.0267
Q20 of clean data (%)	97.27	97.46	97.49	97.51	97.27	97.36
GC of clean data (%)	51.36	50.78	51.73	51.74	50.5	51.21
Total reads	43001618	51683604	56884578	43412048	46062734	49493736
Total mapped	40441636	49084894	53585721	41065739	43941184	47020732
Total mapping ratio (%)	94.05	94.97	94.2	94.6	95.39	95.0
Multiple mapped	4385425	5441656	5524505	3995155	3551354	3707534
Multiple mapping ratio (%)	10.2	10.53	9.71	9.2	7.71	7.49
Uniquely mapped	36056211	43643238	48061216	37070584	40389830	43313198
Uniquely mapping ratio (%)	83.85	84.44	84.49	85.39	87.68	87.51

### Screening of DEGs in the ESW-treatment psoriasis mice

**Fig 2:**
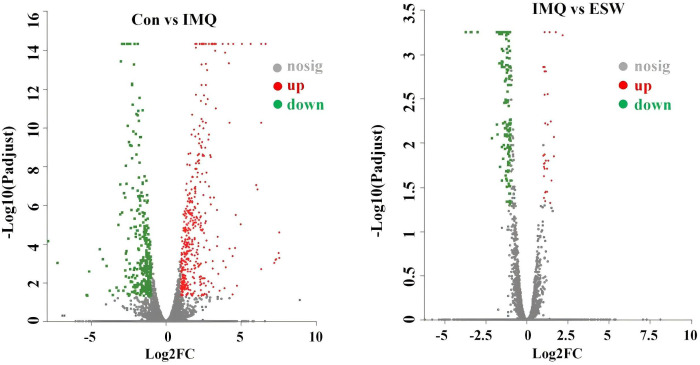
The volcano plots illustrated gene expression differences between Con, IMQ, and ESW groups. Each dot represented an individual gene. Red dots indicated significantly up-regulated genes, green dots represented significantly down-regulated genes, and gray dots denoted genes with no significant changes in expression.

**Fig 3:**
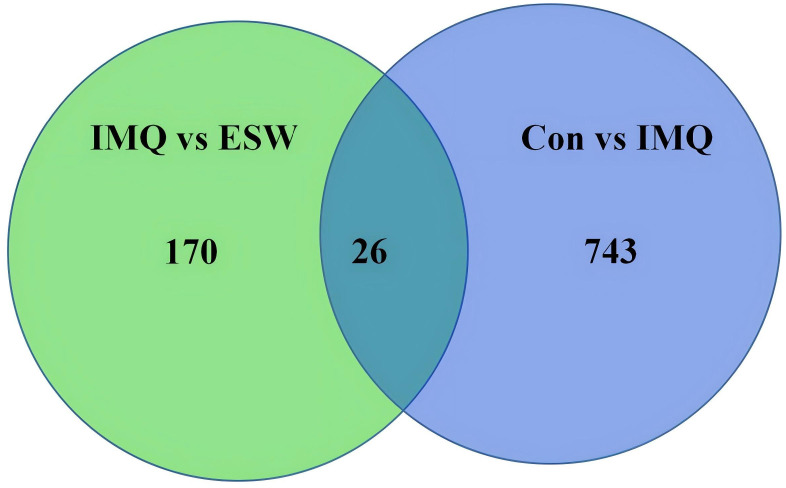
The Venn diagram illustrated the comparison between IMQ vs ESW and Con vs IMQ. The overlapping section in the center indicated the number of genes that were concurrently altered in both contrast groups, which was 26.

**Fig 4:**
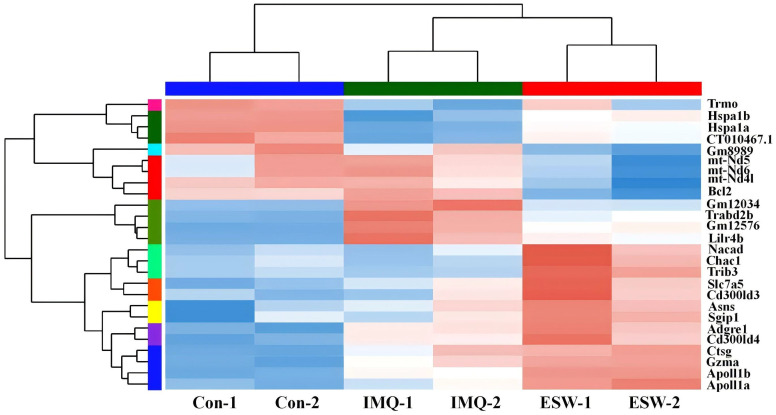
The heatmap illustrated the clustering of 26 differentially expressed genes. Red denoted up-regulated expression, while blue represented down-regulated expression. The intensity of the color corresponded to the magnitude of genes expression changes, with darker shades indicating more significant alterations.

The gene expression across different groups were visualized through volcano-plots (Fig 2). A total of 769 DEGs were identified in IMQ group versus Con group, with 425 upregulated and 344 downregulated. Compared to IMQ group, 196 DEGs were identified in ESW group, including 32 upregulated and 164 downregulated. The Venn diagram showed that there were 26 overlapping genes, among which a total of 170 genes that remarkably upregulated or downregulated in IMQ group were significantly normalized by ESW treatment (Fig 3). The DEGs across different groups were shown in heatmaps (Fig 4). Practically, expression levels of 18 genes among the 26 DEGs were significantly different between IMQ group and ESW group (Table 3). In psoriasis mice without ESW, 13 genes showed higher and 5 showed lower expression (*P* < 0.05). In psoriasis mice with ESW, 5 genes had higher and 13 genes had lower expression (*P* < 0.05).

**Table 3:** Psoriasis-associated genes in the spleen after ESW treatment.

Gene name	Description	IMQ/Con		IMQ/ESW	
		FC	*P*-value	FC	*P*-value
*Slc7a5*	solute carrier family 7	2.66	2.42E-08	2.23	1.64E-05
*mt-Nd5*	mitochondrially encoded NADH dehydrogenase 5	0.37	4.10E-05	0.42	2.17E-05
*mt-Nd6*	mitochondrially encoded NADH dehydrogenase 6	0.41	6.77E-05	0.47	2.00E-04
*mt-Nd4l*	mitochondrially encoded NADH dehydrogenase 4L	0.22	7.66E-13	0.36	5.22E-05
*Trib3*	tribbles pseudokinase 3	5.00	1.86E-07	8.13	1.10E-10
*Bcl2*	B cell leukemia/lymphoma 2	0.44	5.87E-07	0.47	7.75E-06
*Chac1*	cation transport regulator 1	6.16	1.27E-07	10.61	4.66E-11
*GzmA*	granzyme A	4.55	1.05E-12	2.12	2.13E-11
*Asns*	asparagine synthetase	2.91	4.96E-09	2.08	6.93E-05
*Gm8989*	predicted gene 8989	0.18	3.02E-12	0.38	0.001
*Adgre1*	adhesion G protein-coupled receptor E1	3.86	1.51E-12	2.03	8.98E-05
*CTSG*	cathepsin G	5.73	4.56E-22	2.07	0.001
*Sgip1*	SH3-domain GRB2-like interacting protein 1	2.15	7.32E-05	2.03	1.20E-04
*Apol11a*	apolipoprotein L 11a	3.12	3.32E-07	2.50	2.84E-05
*Apol11b*	apolipoprotein L 11b	2.85	1.37E-11	2.07	1.70E-06
*Nacad*	NAC alpha domain containing	2.85	1.77E-06	2.91	7.13E-06
*Cd300ld4*	CD300 molecule like family member D4	5.32	3.06E-12	2.41	8.89E-05
*Cd300ld3*	CD300 molecule like family member D3	4.87	6.17E-08	3.51	8.77E-05

### GO functions analysis of DEGs

**Fig 5:**
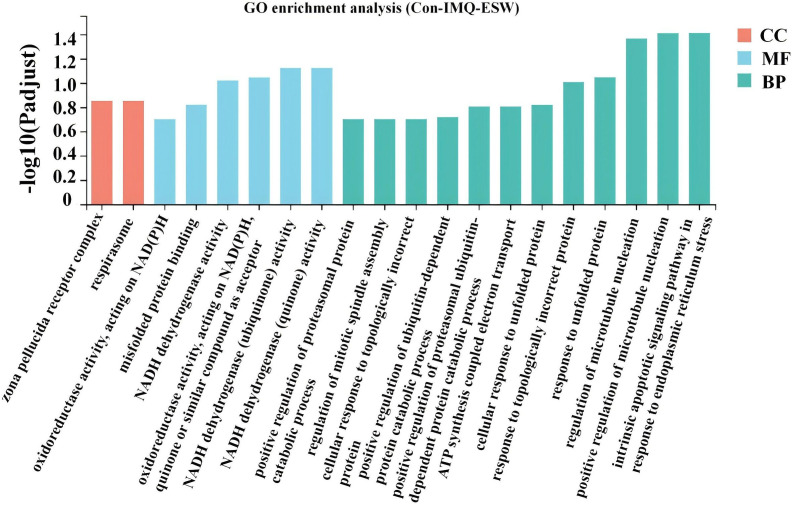
The bar graph presented the results of GO enrichment analysis after ESW treatment for psoriasis. It highlighted significant changes in mRNA levels associated with enriched GO terms, categorized into three main groups: CC, MF, and BP. The y-axis represented the -log10 (P-adjust), indicating the statistical significance of enrichment, with higher values denoting greater significance.

To fully investigate the potential biological roles of DEGs in splenic tissues, GO, a gene function classification system, was used for further description of the identified DEGs properties. The 26 DEGs were categorized into three sections on the basis of their functions and biological pathways, containing BP, CC and MF. The biological processes, cellular components, and molecular activities of DEGs that were most dramatically enriched in psoriasis mice after ESW intervention included the intrinsic apoptotic signaling pathway in reaction to endoplasmic reticulum stress, positively regulating microtubule nucleation, regulation of microtubule nucleation, respirasome, zona pellucida receptor complex, and NADH dehydrogenase activity (Fig 5).

### KEGG pathways analysis of DEGs

**Fig 6:**
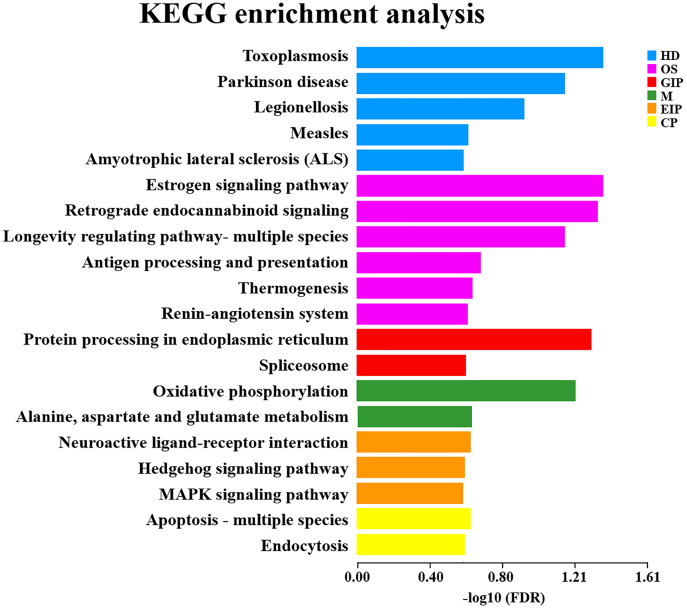
The bar graph illustrated the enrichment of DEGs in various KEGG pathways after ESW treatment for psoriasis. The y-axis listed the enriched pathways, and the x-axis represented the -log10(FDR), where a smaller false discovery rate (FDR) corresponded to a greater -log10(FDR) value, indicating higher significance. Different colors denoted the six branches of the KEGG classification: Human Diseases in blue, Organismal Systems in purple, Genetic Information Processing in red, Metabolism in green, Environmental Information Processing in orange, and Cellular Processes in yellow.

The KEGG database, rich in pathway information, was beneficial to elucidate the overall biological functions of genes. Twenty of the most significantly enriched pathway items were selected and displayed in a scatter plot based on KEGG analysis. The DEGs that were reversed by ESW treatment were considered potential therapeutic targets of ESW in this study. These DEGs were most significantly involved in six first categories, including human disease, organic system, processing of ambient information, metabolism, genetic information and cellular information. Furthermore, the top second-category KEGG pathways contained toxoplasmosis, Parkinson disease, legionellosis, estrogen signaling pathway, retrograde endocannabinoid signaling, lifespan-regulating pathway-multiple species, oxidative phosphorylation, spliceosome, endoplasmic reticulum protein processing, alanine, aspartate and glutamate metabolism, and neuroactive ligand-receptor interaction, hedgehog signaling pathway, MAPK signaling pathway, apoptosis – multiple species, endocytosis, antigen processing and presentation, thermogenesis (Fig 6). These findings demonstrated that the beneficial effects of ESW on IMQ-induced psoriasis in mice may be associated with regulation of the processes.

### Validation of the RNA-seq results by qPCR

**Fig 7:**
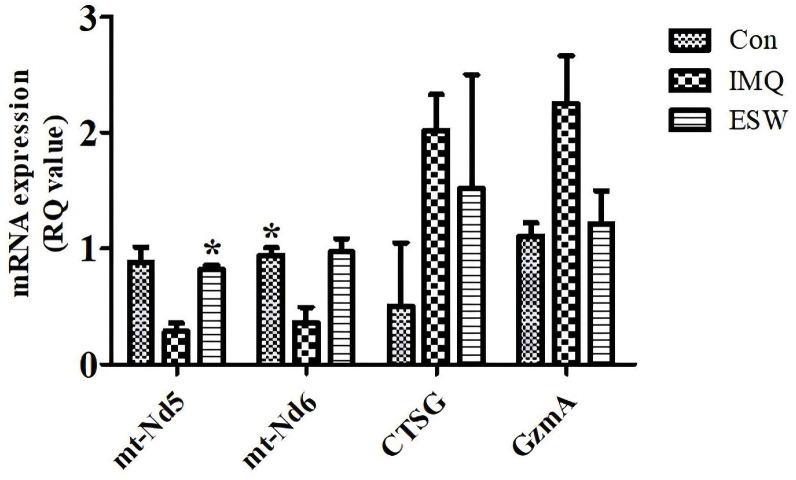
The relative mRNA expression levels of selected genes as determined by quantitative real-time PCR (qPCR). The y-axis represented mRNA expression normalized to an internal control, expressed as RQ (Relative Quantity) values. The genes analyzed included *mt-Nd5*, *mt-Nd6*, *CTSG*, and *Gzma*. Different patterns of bars represented the three experimental groups: Con, IMQ and ESW. Error bars indicated standard deviation, and asterisks denoted statistically significant differences compared to Con group (*P < 0.05, ** *P* < 0.01).

To validate the accuracy and repeatability of RNA-seq findings, we performed qPCR on 4 DEGs with the results presented in Fig 7. *GzmA* and *CTSG*, which are involved in the process of neuroactive ligand-receptor interaction pathway, were significantly upregulated in IMQ group compared to Con group, and downregulated with ESW treatment. Additionally, *mt-Nd5* and *mt-Nd6*, associated with NADH dehydrogenase activity, oxidative phosphorylation, showed a similar pattern of regulation. The qPCR data corroborated the RNA-seq findings, thus confirming the reliability of the high-throughput sequencing results. These findings underscored the potential of ESW in modulating specific gene expression pathways relevant to psoriasis.

### PPI network analysis

**Fig 8:**
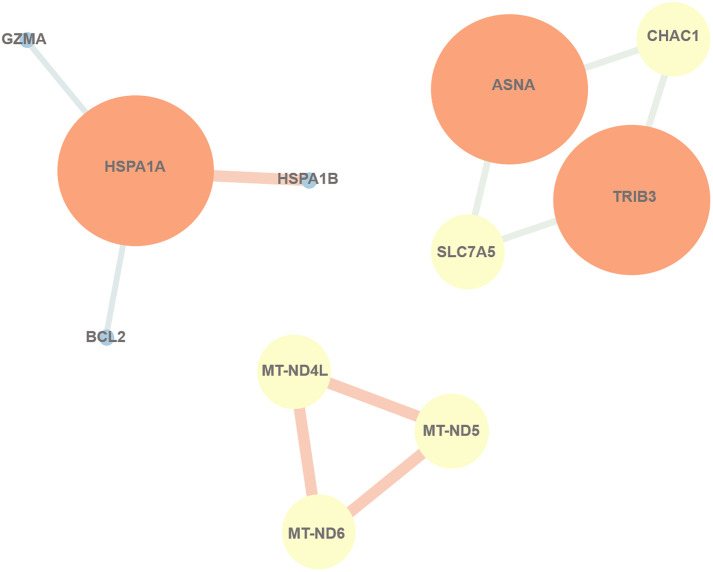
The PPI network of DEGs was constructed using Cytoscape software. The network visualized the interactions between key proteins, with nodes representing individual genes and edges indicating the interactions between them. The color coding differentiates between various protein groups or functional categories, providing insights into the molecular relationships within the studied biological system.

To analyze the functional contributions of the DEGs, PPI network analysis was performed using STRING and Cytoscape. The result demonstrated that *Asns* was related to *Chac1*, *Trib3*, and *Slc7a5*, while *Hspa1a* was associated with *Hspa1b*, *Bcl-2* and *GzmA*. Moreover, there were observed connections among *mt-Nd4l*, *mt-Nd5*, and *mt-Nd6* genes, as depicted in Fig 8.

## Discussion

Although the variety of available treatment methods, psoriasis remains incurable. TCM plays a significant role in the treatment of psoriasis through multi-channel and multi-target. Transcriptomics, the primary tool for studying TCM, can identify signal pathways and multiple target genes. In this study, we explored the underlying molecular processes of ESW alleviating IMQ-induced psoriasis-like skin inflammation in mice by using RNA-seq. Among the 26 DEGs, *Hspa1a* and *Hspa1b* had the most connections with the enriched pathways, including antigen processing and presentation, MAPK signaling pathway, unfolded protein response, estrogen signaling pathway, the endoplasmic reticulum protein processing, and others. HSPA1A and HSPA1B belong to the heat shock proteins (HSPs) family, which are cytoprotective substances produced in response to a range of stressful stimuli such as heat, microbial infections, and inflammatory mediators. *Hspa1a* and *Hspa1b* are related to B cells, which are integral to the immune system [13]. Epitopes derived from HSP70 can interact with the B cell receptor, leading to the production of anti-HSP70 antibodies [14]. HSPs have emerged as therapeutic target in psoriasis, with HSP70 molecules being crucial for the immune response because they act both as chaperones and inducers of proinflammatory cytokine release [15]. HSP70 as a target for treating psoriasis has been demonstrated in the IMQ mouse model and related assays *in vitro* [16]. The tolerance to self-antigens of psoriasis can be broken by chemicals interfering with antigen processing and presentation [17]. It is hypothesized that HSPs may similarly disrupt this tolerance, thereby influencing disease progression, although the specific mechanisms remain to be elucidated. Furthermore, HSPs are related to the MAPK signaling pathway. The corticotropin-releasing hormone (CRH) in human keratinocytes regulates IL-18 production through the MAPK signaling pathway [18]. Similarly, calcitonin gene-related peptide (CGRP)regulates the expression of VEGF through the ERK1/2 MAPK signaling pathway [19]. CCN1, a novel proinflammatory factor and extracellular protein, increases IL-1β production via p38 MAPK signaling, thereby promoting inflammation in psoriasis [20]. Moreover, recent studies have shown that TCM can relieve psoriasis inflammation through MAPK signaling pathway. For instance, Longkui Yinxiao Soup [21] and the herb pair *Cimicifuga foetida* L. and *Rheum palmatum* L have been shown to exert anti-inflammatory effects via this pathway [22]. These findings highlight the potential therapeutic applications of TCM in modulating inflammatory responses in psoriasis through MAPK signaling. The unfolded protein response (UPR) is an intracellular stress response mechanism primarily triggered by the endoplasmic reticulum (ER). Its central function is to address the accumulation of misfolded or incorrectly folded proteins within the ER, thereby maintaining protein homeostasis and normal cellular function. The downregulation of UPR markers, such as GRP78/Bip and HRD1, in psoriasis vulgaris has been well documented [23,24]. This impairment of the UPR is thought to contribute to the abnormal keratinocyte differentiation and hyperproliferation characteristic of psoriasis. Our findings, which showed enrichment of the UPR pathway in psoriasis, were consistent with these reports and further highlighted the potential significance of UPR dysfunction in the pathogenesis of psoriasis. In addition to *Hspa1a* and *Hspa1b*, cation transport regulator homolog 1 (*Chac1*) is also related to UPR. *Chac1* is an ER stress-inducible gene encoding a γ-glutamyl cyclotransferase that degrades glutathione, playing a role in oxidative stress and apoptosis [25]. Importantly, *Chac1* is also correlated with the inflammatory response and NF-κB signaling pathway [26]. Recent research has highlighted that CHAC1 may influence the development of psoriasis by regulating ferroptosis, suggesting its potential as a biomarker for this condition [27]. Our findings further supported the involvement of *Chac1* in UPR-related processes and its relevance to psoriasis pathogenesis.

Apart from above-mentioned pathways, *Hspa1a* and *Hspa1b* are also implicated in the estrogen signaling pathway and protein processing in ER. In psoriasis, the binding sites of estrogen receptor‑1 (ESR1) are enriched with genes possessing anti‑apoptotic functions [28]. Estrogen receptor β (ERβ) has been shown to reduce inflammation and suppresses the activation of the NF-κB pathway [29]. Furthermore, estradiol may increase skin flap survival by preventing neutrophil infiltration and reducing the expression of p38‐MAPK [30]. Interestingly, estrogen may also exacerbate psoriasis-like skin inflammation possibly by acting on ERβ [31]. Another key player in autoimmune diseases is endoplasmic reticulum aminopeptidase 1 (ERAP1), a member of the M1 family of aminopeptidases. ERAP1 is crucial for processing antigenic peptides in the ER, where it trims their N-termini before they are loaded onto HLA-I molecules for antigen presentation [32]. Intriguingly, in this study, the expression of *Hspa1a* and *Hspa1b* were downregulated in IMQ group compared with Con group. However, this downregulation persisted even after administration of ESW. The reasons for this observation require further investigation.

Interestingly, interruption of the estrogen signaling pathway also affects the expression of *Bcl-2* [33]. Psoriasis is associated with cellular homeostasis disorders, particularly apoptosis. The *Bcl-2* family of genes, which are closely related to apoptosis, participate in the release of cytochrome c from mitochondria and can serve as potential diagnostic markers for psoriasis [34]. In studies involving RP6-65G23.1-knockdown [35] and Wnt5a knockdown cells [36], both cell proliferation and apoptosis were affected, with the expression of Bcl-2 downregulated. Nitidine chloride relieves psoriasis skin lesions and inflammation by inhibiting HaCaT proliferation, inducing S phase cell cycle arrest, and significantly downregulating *Bcl-2*. Additionally, the hedgehog (Hh) signaling pathway is implicated in *Bcl-2*. Activation of the Hh pathway upregulates *Bcl-2*, thereby preventing apoptosis by maintaining mitochondrial membrane integrity [37]. In psoriasis, the Hh signaling pathway is triggered [38]. In the HaCaT cells, recombinant human parathyroid hormone (1–34) modulates proteins expression within the Hh signaling pathway [39], while arturmerone suppresses cell proliferative ability and attenuates inflammatory cytokine expression by inactivating Hh pathway [40]. Herbal products, which have fewer side effects, have been used as alternative treatments for psoriasis, partly through inhibition of the Hh signaling pathway [41]. Our study demonstrated that *Bcl-2* expression was deregulated in IMQ-induced mice, a phenomenon that was reversed after ESW administration. This result suggested that ESW may reduce the psoriasis symptoms by regulating *Bcl-2* expression.

The three differentially expressed genes, *mt-ND4L*, *mt-ND5* and *mt-ND6*, are mitochondrial genes encoding NADH dehydrogenase (mt-ND) and involved in some pathways, including NADH dehydrogenase activity, oxidative phosphorylation, and thermogenesis. Disfunction of the mitochondrial respiratory chain and decreased ATP production can result from *mt-Nd* gene failure [42]. For instance, the mutations of *mt-ND4L* and *mt-ND6* can impair the function of the oxidative phosphorylation system (OXPHOS) enzymes, subsequently affecting the production of ATP [43]. It has been reported that the expression of serum extracellular mitochondrial DNA (mt DNA) and mitochondrial regulatory proteins in psoriatic skin is related to inflammation and keratinocyte apoptosis [44]. Certain mtDNA variants have been shown to play a protective role in psoriasis [45]. Following the integration of signals from metabolism and proteostasis on the mitochondrial surface, the NADH dehydrogenase Nde1 executes cell death [46]. Mitochondria provides energy for cells through the OXPHOS. The largest enzyme complex in OXPHOS, mitochondrial complex I, helps to produce the proton-motive force (PMF), which is used to oxidize NADH and produce ATP [47]. The hyperproliferation of psoriatic keratinocytes is heavily dependent on the production of energy by OXPHOS. Th17 effector cells depend on OXPHOS for energy and cytokine production. Thus, inhibition of OXPHOS can reduce the severity of psoriasis. Targeting *Nrf2* to decrease mitochondrial OXPHOS-driven oxidative stress in keratinocytes has been reported to attenuate IL-17A-induced psoriasis by regulating the bioenergetic metabolism [48]. Gonadal white AT in a mouse dermatitis model showed an impaired thermogenesis ability due to systemic inflammation. Our study demonstrated that IMQ downregulated *mt-ND4L*, *mt-ND5*, and *mt-ND6*, while ESW administration could reverse this effect, indicating that ESW may improve psoriasis inflammation by regulating mitochondria and energy metabolism.

The neuroactive ligand-receptor interaction pathway was one of the significantly enriched pathways in our analysis. The miRNA-146a plays a significant part in psoriasis progression, partially by regulating the neuroactive ligand-receptor interaction pathway. The differentially expressed genes Granzyme A(*GzmA*) and Cathepsin G (*CTSG*) are involved in this pathway. *GzmA*, a kind of immune-related genes, belongs to the family of serine proteases and can promote pro-inflammatory. The expression of *GzmA* is elevated in CD8 T cells isolated from psoriatic lesions [49] *GzmA* also targets the endoplasmic reticulum-associated SET complex, leading to apoptotic cell death [50]. CTSG is a 26-kDa serine protease expressed during the development stage of promyelocyte. It is involved in host defense and immune response mediated by neutrophils [51], and its inhibition can decrease neutrophilic infiltration and the expression of IL-1β [52]. Correspondingly, the expressions levels of *GzmA* and *CTSG* were increased in the IMQ group and decreased in ESW group.

The results of PPI network analysis showed interactions among *mt-Nd41*, *mt-Nd5* and *mt-Nd6* genes. *Hspa1a* was associated with *Hspa1b*, *Bcl-2* and *CTSG*. Notably, the gene Asparagine synthetase (*Asns*) was directly linked to *Trib3*, *Slc7a5*, and *Chac1*. *Asns* catalyzes the conversion of aspartate and glutamine to asparagine and glutamate. It is related to apoptotic suppression, protein biosynthesis, and mTORC1 activationand its expression can be regulated by the UPR pathways. *Trib3*, a mammalian tribbles homolog, has been shown to improve metabolism when it is silenced *in vivo* [53]. The inhibition of *Trib3* expression ameliorated ER stress to significantly ameliorates apoptosis [54]. Moreover, *Trib3* knockdown impacts inflammatory processes via inhibiting *Wnt5a* expression and NF-κB phosphorylation [55]. *Trib3* is also involved in the activation of several signaling pathways, including MAPK pathways [56]. SLC7A5, also known as L-type amino-acid transporter 1 (LAT-1), a system L-type transporters, is crucial for cell maintenance and proliferation. It provides cells with essential amino acids. In autoimmune diseases, the *Slc7a5*-mTORC1 pathway may offer a new therapeutic approach, LAT1 deletion or inhibition effectively regulates IL-23 and IL-1β-induced PI3K/AKT/mTOR activation [57]. In our study, we found that *Asns*, *Trib3*, *Slc7a5* and *Chac1* were regulated by ESW, indicating a potential connection between these genes and ESW.

This work elucidated the gene-level mechanisms of *P. clypearia* in the treatment of psoriasis by transcriptomics. With its multi-component and multi-target advantages, *P. clypearia* acted on various signaling pathways and gene targets, in contrast to conventional medications, representing TCM holistic view to disease treatment. The omics method offers a valuable reference for the modernization of TCM research as well as an example for fully comprehending the pharmacological effects of medications. Future research will incorporate multi-omics data, such as proteomics and metabolomics, to analyze gene functions and interactions in depth, thereby creating a more comprehensive molecular regulatory network. Furthermore, the biological functions of key genes and their specific mechanisms in ESW treatment psoriasis will be further validated through the design of gene knockout or overexpression experiments.

## Conclusion

Based on TCM theory of the lung governing the skin and fur and the therapeutic principles of clearing heat and cooling blood, we had confirmed for the first time that *P. clypearia*, an herbal medicine used for treating respiratory infections, could ameliorate psoriasis in IMQ-induced mice [12]. In this study, transcriptomics was employed for the first time to explore the underlying molecular mechanisms of ESW from *P. clypearia* in alleviating IMQ-triggered psoriasis-like cutaneous inflammation in mice and 26 DEGs were identified. Among the 26 DEGs, the most of genes that showed increased expression in IMQ group exhibited a reversal after the administration of ESW. The DEGs were investigated using GO, KEGG, PPI network analysis and verified by qPCR. The results indicated that *P. clypearia* may affect the energy metabolism, cell growth and apoptosis by regulating genes such as *Hspa1a*, *Hspa1b*, mt-Nd4l, *mt-Nd5*, *mt-Nd6*, *Bcl-2*, *Asns*, *Trib3*, *GzmA*, *CTSG*, etc, thereby mitigating psoriasis-related inflammation. Our research results provided a theoretical basis for the treatment of psoriasis with *P. clypearia*, but the specific molecular mechanisms at the cellular and molecular levels was still needed to be further studied. This study explored the prospect of these genes for biomarkers or therapeutic targets development, facilitating the translation of basic research into clinical applications and offering new theoretical foundations and practical directions for the precise diagnosis and treatment of psoriasis.
